# Cost-effectiveness of interventions for increasing the possession of functioning smoke alarms in households with pre-school children: a modelling study

**DOI:** 10.1186/1471-2458-14-459

**Published:** 2014-05-16

**Authors:** Pedro Saramago, Nicola J Cooper, Alex J Sutton, Mike Hayes, Ken Dunn, Andrea Manca, Denise Kendrick

**Affiliations:** 1 Centre for Health Economics, University of York, York, UK; 2 Department of Health Sciences, University of Leicester, 2nd Floor Adrian Building, University Road, Leicester LE1 7RH, UK; 3 Child Accident Prevention Trust, Canterbury Court (1.09), London, UK; 4 iBID, South Manchester University Hospital, Manchester, UK; 5 Division of Primary Care, University of Nottingham, Nottingham, UK

**Keywords:** Cost-effectiveness analysis, Smoke alarms, Decision model, Fire-related injuries, Child home injuries

## Abstract

**Background:**

The UK has one of the highest rates for deaths from fire and flames in children aged 0–14 years compared to other high income countries. Evidence shows that smoke alarms can reduce the risk of fire-related injury but little exists on their cost-effectiveness. We aimed to compare the cost effectiveness of different interventions for the uptake of ‘functioning’ smoke alarms and consequently for the prevention of fire-related injuries in children in the UK.

**Methods:**

We carried out a decision model-based probabilistic cost-effectiveness analysis. We used a hypothetical population of newborns and evaluated the impact of living in a household with or without a functioning smoke alarm during the first 5 years of their life on overall lifetime costs and quality of life from a public health perspective. We compared seven interventions, ranging from usual care to more complex interventions comprising of education, free/low cost equipment giveaway, equipment fitting and/or home safety inspection.

**Results:**

Education and free/low cost equipment was the most cost-effective intervention with an estimated incremental cost-effectiveness ratio of £34,200 per QALY gained compared to usual care. This was reduced to approximately £4,500 per QALY gained when 1.8 children under the age of 5 were assumed per household.

**Conclusions:**

Assessing cost-effectiveness, as well as effectiveness, is important in a public sector system operating under a fixed budget restraint. As highlighted in this study, the more effective interventions (in this case the more complex interventions) may not necessarily be the ones considered the most cost-effective.

## Background

Child injuries have been identified by the World Health Organization as a growing global public health problem [1]. There is a need globally to increase awareness of the problem and promote effective ways of reducing the incidence and severity of childhood injuries. The majority of injuries in young children occur in the home, with fire-related injuries being particularly important in terms of resultant disabilities, deaths and costs incurred [2,3]. Furthermore, the UK has one of the highest rates for deaths from fire and flames in children aged 0–14 years compared to other high income countries [4]. In 2011–12 the Fire and Rescue Services in Great Britain attended over 44,300 domestic fires [5]. Within the same period 11 fatalities were estimated to have happened as a result of accidental fires in the home for the 0–4 age group [6]. Fires detected by smoke alarms tend to be discovered more rapidly and are associated with a reduced risk of death and property damage [7-9]. Publicity campaigns, such as Fire Kills [10], have been conducted in the UK in an attempt to increase the number of households which have ‘functioning’^a^ smoke alarms fitted but few evaluations have been conducted to assess their impact on fatal and non-fatal injuries of young children in terms of their lifetime costs and effects (i.e. quality of life). This is of particular interest because children under the age of 3 years are at the highest risk of burn mortality both with and without smoke inhalation injury [11].

Four studies [12-15] to date have evaluated the cost-effectiveness (using a decision model-based analyses) of schemes to promote the installation of functioning smoke alarms in the home, with only one of these focusing on the costs and benefits to children [14]. The economic evaluation by Pitt *et al.*[14], commissioned by the National Institute for Health and Care Excellence (NICE), was primarily based on Ginnelly *et al.*[13] but the analysis was targeted towards reducing unintentional injuries from house fires in children under 15 years of age. This decision model-based analysis found the installation of free smoke alarms to be cost effective. Three determinants were found to be the main drivers of the results obtained by Pitt *et al.*; these include the existing prevalence of use of safety devices, the proportion of households that choose to participate in a programme, and the proportion that correctly install or use any devices provided.

The aim of our analysis is to develop a decision analytic model to evaluate the cost-effectiveness of having functioning smoke alarms in households with children less than 5 years of age. We extend the analysis by Pitt *et al.*[14] to include effectiveness data for all previously trialled interventions (i.e. a combination of education, free or low cost equipment giveaway, equipment fitting and/or home safety inspection) to increase uptake of functioning smoke alarms in households and hence, reduce fire-related fatal and non-fatal injuries in children. The cost-effectiveness of all the different interventions is compared.

## Methods

### Decision problem

The cost-effectiveness analysis compared a range of different intervention strategies developed to increase uptake of functioning smoke alarms in households and hence, reduce fire-related fatal and non-fatal (minor, moderate or severe^b^) injuries in children. These were identified from a recently published mixed treatment comparison meta-analysis [16] as: (1) Usual care (UC); (2) Education (E); (3) Education + free/low cost equipment (E + FE); (4) Education + free/low cost equipment + home safety inspection (E + FE + HI); (5) Education + free/low cost equipment + fitting (E + FE + F); (6) Education + home safety inspection (E + HI); (7) Education + free/low cost equipment + fitting + home safety inspection (E + FE + F + HI).

We considered a hypothetical population of newborns and evaluated the impact that living in a household with or without a functioning smoke alarm during the first 5 years (0–4 years of age) of their life would have on their overall lifetime costs and quality of life. We constructed a 3-stage mathematical model (details below) to estimate the lifetime QALYs and costs of the interventions from a public sector perspective^c^ (which includes UK National Health Service (NHS) and Personal Social Care Services (PSS) costs, together with other public sector costs), discounted at the standard annual rate of 3.5% [17]. Findings were expressed in terms of incremental cost-effectiveness ratios (ICERs) and probabilities of alternative interventions being cost-effective at different decision-makers’ cost per additional QALY thresholds [18].

### Decision model

#### ***Model structure***

**Figure 1:**
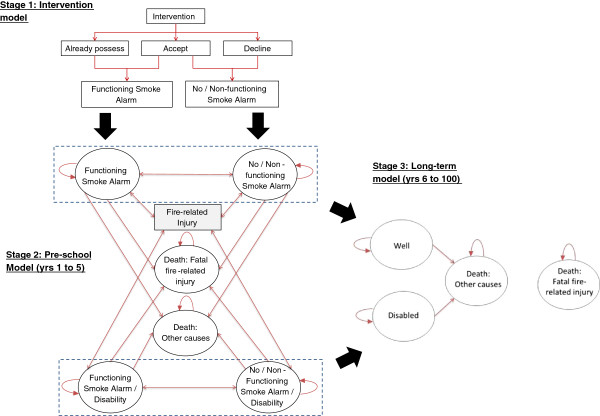
Schematic of the model structure split into 3 stages.

In developing our model we used the principles for good modelling practice and design set out proposed by Philips *et al.*[19] together with the NICE public health methods guidance [20,21].We constructed our model in the software package R version 2.15.1 [Copyright © 2012 The R Foundation for Statistical Computing] and assessed it by Monte Carlo (MC) simulation. Figure 1 illustrates diagrammatically our 3 stage decision model.

Firstly, the effectiveness of an intervention strategy to increase the possession of smoke alarms in households was analysed using a decision tree structure. Results of a previous synthesis of evidence on effectiveness of interventions to promote smoke alarm ownership and function [16] were used to inform this model section. This model accounted for existing prevalence of installed functioning smoke alarms and uptake rate for the intervention amongst those in the modelled population who did not have a functioning smoke alarm installed. This decision tree is referred to as the *intervention model* and was also used to estimate the costs of implementing the interventions under consideration. In this initial part of the model the household: i) may already have a functioning smoke alarm (baseline probability a household having a functioning smoke alarm); or ii) may not have a functioning smoke alarm, accepts the intervention and becomes an enabled functioning smoke alarm household after intervention; or iii) may not have a functioning smoke alarm, accepts the intervention but does not become an enabled functioning smoke alarm household after the intervention; or, finally, iv) may not have a functioning smoke alarm and does not accept the intervention. Households in situations i) and ii) will start the next model stage in the ‘Functioning smoke alarm’ state while households in iii) and iv) will start in the ‘No/Non-functioning smoke alarm’ state.

The second stage of our model, referred to as the *pre-school model*, used a Markov state-transition structure to model the outcomes of fire-related injuries (i.e. minor, moderate and severe) and fatalities of children during the pre-school period (aged 0 to 4). It used the outputs from the intervention model as its primary inputs. The progress between model states is conditional on the occurrence of fires in the household, and on the consequences for the child (fire-related injuries). The model also considers the possibility of the safety equipment ceasing to function and, in the case of it failing to function, that it is repaired. This is achieved with the introduction of a decay/repair factor, which establishes the transition rates, from ‘functioning’ to ‘no/non-functioning’ equipment and vice-versa. This factor affects, at any cycle, the probability of the household having a ‘functioning smoke alarm’ in the following cycle (year).

Stage 3 of our model, referred to as the *long-term model*, uses another Markov state-transition structure to model both the costs and health effects of any fire-related injuries incurred during the pre-school years over the individual’s lifetime. For the Markov models, used in stages 2 and 3 of the process, Figure 1 presents the key health states together with possible transitions between them during each cycle. We used yearly cycle duration and ran our model for the equivalent of 100 years (5 years in stage 2 and 95 in stage 3, respectively) by which time most of the people from our child population had died. By attributing costs (inflated to 2012 prices) and quality of life weights to each state, total costs and QALYs were established for each of the different interventions strategies.

All evidence used to inform the base-case model, together with distribution information where applicable, are presented in Additional file 1: Tables S1 and S2. Where possible, input parameters were informed by UK based data. A summary of the base case methodological assumptions is outlined in Table 1 below.

**Table 1:** Summary of the base case

**Element of assessment**	**Base case**
Type of economic evaluation	Cost-effectiveness analysis
Perspective on costs	Public sector, including the NHS and PSS
Perspective on outcomes	All health effects on individuals
Evidence on outcomes	Simultaneous synthesis of evidence of multiple interventions
Measure of health effects	Quality Adjusted Life Years (QALYs)
Main source of data for measurement of health related quality of life (HRQL)	Reported directly by patients (Medical Care Research Unit, University of Sheffield: *Long Term Health and Healthcare outcomes of Accidental Injury study (HALO).* Unpublished report for the Department of Health))
Source of preference data for valuation of changes in HRQL	Representative sample of the public (UK Population norms [22])
Discount rate	An annual rate of 3.5% was used on both costs and health effects
Equity weighting	An additional QALY has the same weight, regardless of the characteristics of the individuals who gain the health benefit
Size of the cohort simulated	100,000
Time horizon	100 years - until population all dead in order to account for all outcomes

### Main modelling assumptions

As with any model, simplifications and assumptions are required. In this model-based analysis the following were assumed:

i) The possession of ‘functioning’ smoke alarms in the household is a surrogate/intermediate outcome linked to the final endpoint of reduction in risk of injury/death in the household due to fire. This relationship was populated by a range of evidence [2,6,7];

ii) Probability of a household accepting an intervention is assumed the same across all interventions due to a lack of information on the acceptance of the different programmes;

iii) Benefit of a household having a functioning smoke alarm accrues to a single child aged 0 to 4 years of age. It ignores potential (positive or negative) spill over effects on sibling(s) and/or parent(s) living in the same household. This may be a conservative assumption, as multiple people could benefit from a smoke alarm;

iv) Probability of a future fire-related injury is assumed not to be dependent on previous fires or fire-related injury, and remains constant throughout the relevant model timeframe (i.e. 5 years for part 2 of the model). This assumption is common to most Markov models and implies that a household’s awareness of the risk of fires and fire-related injury remains the same, irrespective of whether a previous event occurred; and

v) Only allows for one fire or fire-related injury in a single cycle (i.e. 1 year).

### Uncertainty

Our model took account of uncertainty around the input parameter point estimates. The effectiveness evidence synthesis results were used in the decision model through the 5,000 Markov chain Monte Carlo (MCMC) posterior samples (extracted from the Convergence Diagnostic and Output Analysis WinBUGS output (CODA)). Evidence to inform other model parameters were identified from the literature and we defined a probability distribution for each on the basis of its point estimate and standard error (Additional file 1: Tables S1 and S2). We probabilistically evaluated the decision model by performing 5,000 MC simulations, which randomly selects a value for all parameters from their respective distributions. The number of simulations performed in the decision model was conditional to the number of MCMC simulations in the synthesis analysis. We calculated the mean costs and mean QALYs by averaging across all 5,000 MC simulations.

We performed one-way sensitivity analyses on the following parameters to test the robustness of our results to the model assumptions and data sources.

SA1 Prevalence of smoke alarms in households reduced from 80% to 50% [13];

SA2 Probability of accepting the intervention reduced from 90% to 50% [13];

SA3 Decay of safety equipment reduced from 0.1 to zero;

SA4 Children per household increased from 1 to 1.8 (i.e. the national average [23]); and

SA5 Same probability of injury following a fire for ‘functioning’ and ‘non-functioning’ smoke alarm households, 0.91 [2].

## Results

### Base-case analysis

In our base case analysis, from the set of seven interventions being evaluated, Strategy (3) E + FE was identified to have the lowest estimated ICER when compared to usual care (UC) with £34,200 per QALY gained (Table 2). From the group of 7 interventions being evaluated, 4 were either dominated or extendedly dominated (i.e. having higher costs or higher ICERs than more effective interventions, respectively) by two interventions - strategy (3) E + FE and strategy (7) E + FE + F + HI.

**Table 2:** Base-case cost effectiveness results (probabilistic analysis)

**Intervention**	**Expected QALYs**	**Expected Costs (£s)**	**Incremental QALYs**	**Incremental Costs (£s)**	**ICER (£s per QALY)**	**Probability CE (£30,000)**	**Probability CE (£50,000)**
(1) UC	25,056.393	19,317	----	----	----	0.619	0.312
	(25039.06 to 25073.8)	(7850 to 40561)					
(2) E	25,056.401	20,055	----	----	Extendedly	0.000	0.001
	(25039.07 to 25073.81)	(8750 to 41093)			dominated		
(3) E + FE	25,056.416	20,094	0.023	777	34,200	0.381	0.687
	(25039.09 to 25073.81)	(9193 to 40546)					
(4) E + FE + HI	25,056.416	22,091	----	----	Dominated	0.000	0.000
	(25039.09 to 25073.82)	(11047 to 42710)					
(5) E + FE + F	25,056.416	21,638	----	----	Dominated	0.000	0.000
	(25039.09 to 25073.81)	(10654 to 42219)					
(6) E + HI	25,056.403	21,991	----	----	Dominated	0.000	0.000
	(25039.08 to 25073.81)	(10673 to 43168)					
(7) E + FE + F + HI	25,056.417	23,596	0.001	3,502	3,466,635	0.000	0.000
	(25039.09 to 25073.82)	(12021 to 44319)					

Data are expected QALY (95% credibility interval) and expected costs (95% credibility interval) per 1,000 households. (1) UC = usual care; (2) E = education; (3) E + FE = education plus low cost/free safety equipment; (4) E + FE + HI = education plus low cost/free safety equipment plus home inspection; (5) E + FE + F = education plus low cost/free safety equipment plus fitting; (6) E + HI = education plus home inspection; (7) E + FE + F + HI = education plus low cost/free safety equipment plus fitting plus home inspection. Probability CE = probability that intervention is cost effective at a £30,000/£50,000 threshold value. QALYs = quality-adjusted life years.

**Figure 2:**
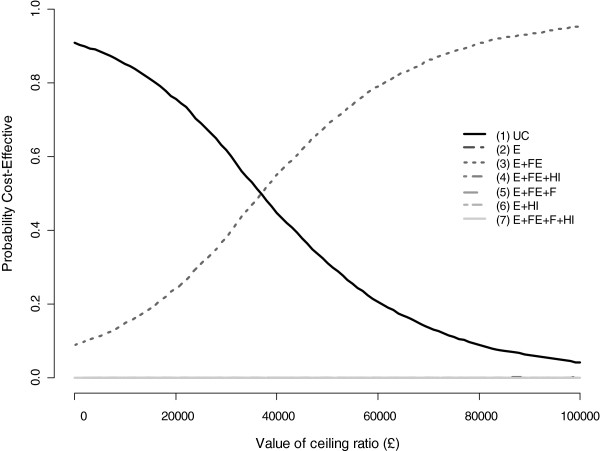
Cost effectiveness acceptability curves.

Figure 2 graphs the probability of the alternative interventions being cost effective. It depicts the typical ‘ogive’ shape of the cost-effectiveness acceptability curves. At a threshold value of £30,000 per QALY gained, usual care has the highest probability of being cost effective (0.62). However, when this threshold value is increased to £50,000, strategy (3) E + FE*,* has the highest probability of being cost effective (0.69). This shows a high level of uncertainty in decisions within the £30,000-£40,000 threshold range.

### Sensitivity analysis

A range of sensitivity analyses varying the base-case assumptions and inputs, as outlined in the methods section, were implemented (Table 3). Following Ginnelly *et al.*[13], scenario *SA1* assessed the impact of a reduction of the prevalence of smoke alarms in UK households from 80% to 50%. This analysis resulted in (1) Usual care being considered the only cost effective intervention (probability of being cost effective at £30,000 of 0.99), as other interventions were associated with higher costs and in order for them to be adopted, decision makers needed to be ‘willing to pay’ or displace large amounts of funds.

**Table 3:** Sensitivity analysis results

Intervention*	**Expected QALYs**	**Expected costs (£s)**	**Incremental QALYs**	**Incremental costs (£s)**	**ICER (£s)**	**Probability CE (£30,000)**	**Probability CE (£50,000)**
SA1: prevalence of smoke alarms in households of 50%							
(1) UC	25,056.054	20,813	----	----	----	0.987	0.984
	(25038.86 to 25073.69)	(8337 to 43726)					
(2) E	25,056.070	23,732	0.016	2,919	180,400	0.000	0.000
	(25038.88 to 25073.71)	(11327 to 46646)					
(3) E + FE	25,056.079	25,715	0.009	1,983	225,545	0.013	0.015
	(25038.88 to 25073.72)	(13029 to 48245)					
(7) E + FE + F + HI	25,056.081	37,863	0.002	12,148	5,955,269	0.000	0.000
	(25038.89 to 25073.72)	(18872 to 61155)					
SA2: probability of accepting the intervention of 50%							
(1) UC	25,056.159	19,470	----	----	----	0.238	0.086
	(25038.67 to 25074.24)	(7948 to 40486)					
(3) E + FE	25,056.177	19,695	0.018	225	12,701	0.762	0.914
	(25038.69 to 25074.26)	(8618 to 39932)					
(7) E + FE + F + HI	25,056.177	21,656	0.000	1,961	3,502,138	0.000	0.000
	(25038.7 to 25074.26)	(10383 to 42046)					
SA3: null decay of safety equipment							
(1) UC	25,056.404	18,839	----	----	----	0.960	0.817
	(25039.07 to 25073.81)	(7684 to 39507)					
(2) E	25,056.413	19,530	0.009	691	80,117	0.038	0.171
	(25039.08 to 25073.82)	(8558 to 39944)					
(3) E + FE	25,056.416	20,094	0.003	564	209,061	0.001	0.012
	(25039.09 to 25073.81)	(9193 to 40546)					
(7) E + FE + F + HI	25,056.417	23,596	0.001	3,502	3,466,635	0.000	0.000
	(25039.09 to 25073.82)	(12021 to 44319)					
SA4: considering 1.8 children per household							
(1) UC	44,349.503	32,867	----	----	----	0.114	0.029
	(44318.77 to 44380.1)	(12272 to 71150)					
(3) E + FE	44,349.544	33,050	0.041	183	4,456	0.885	0.968
	(44318.82 to 44380.14)	(13428 to 69595)					
(7) E + FE + F + HI	44,349.546	36,531	0.002	3,481	1,923,416	0.000	0.000
	(44318.83 to 44380.14)	(16836 to 73296)					
SA5: same probability of injury for households with functioning and non-functioning smoke alarms							
(1) UC	25,056.511	15,279	----	----	----	0.960	0.943
	(25039.23 to 25073.87)	(6611 to 31524)					
(3) E + FE	25,056.519	16,562	0.008	1,283	154,513	0.040	0.057
	(25039.24 to 25073.88)	(7924 to 32584))					
(7) E + FE + F + HI	25,056.520	20,080	0.001	3,518	9,772,579	0.000	0.000
	(25039.23 to 25073.88)	(10842 to 35798)					

Data are expected QALY (95% credibility interval) and expected costs (95% credibility interval) per 1,000 households. (1) UC = usual care; (2) E = education; (3) E + FE = education plus low cost/free safety equipment; (4) E + FE + HI = education plus low cost/free safety equipment plus home inspection; (5) E + FE + F = education plus low cost/free safety equipment plus fitting; (6) E + HI = education plus home inspection; (7) E + FE + F + HI = education plus low cost/free safety equipment plus fitting plus home inspection. Probability CE = probability that intervention is cost effective at a £30,000/£50,000 threshold value. QALYs = quality-adjusted life years.

*Showing only interventions that were not dominated or extendedly dominated.

A high level of heterogeneity was observed across the trials informing the effectiveness model input parameters with respect to the probability of accepting the interventions. Therefore, scenario *SA2* considers a reduction in the acceptance rate from 90% to 50%, resulting in Strategy (3) E + FE having the highest probability of being cost-effective (0.76) at a £30,000 threshold value. In scenario *SA3*, the probability for decay/repair of the safety equipment for transitions between ‘functioning’ and ‘non-functioning’ equipment and vice-versa was reduced from 0.1 to 0 leading to (1) Usual care having the highest probability (0.96) of being cost effective at £30,000 ceiling ratio.

An increase in the number of children under 5 per household from 1 to 1.8 [23], scenario *SA4*, - and assuming that children are of similar age and suffer the same costs and consequences in the event of a home fire – results in Strategy (3) E + FE having the highest probability of being cost effective (0.89) at the £30,000 threshold; which is intuitive as more children will be protected by the smoke alarm. Finally, scenario *SA5* takes a more conservative approach by considering equivalent child injury probabilities for household fires where ‘functioning’ and ‘no/non-functioning’ smoke alarm are present [2], rather than considering differential injury probabilities as in the base case. As for scenario *SA1*, this analysis showed that ‘active’ interventions are linked to higher estimated ICERs and (1) Usual care is the only cost effective strategy (with probabilities of being cost effective at £30,000 and £50,000 very close to 1).

## Discussion

Assessing the cost effectiveness of alternative strategies is important in a public sector system operating under fixed budget constraints. This study evaluated the cost-effectiveness of alternative interventions to increase the household uptake of ‘functioning’ smoke alarms and, consequently, reduce the number and severity of home fire-related injuries in pre-school children. The results of a previous synthesis of evidence on the effectiveness of interventions of interest [16] were used to populate the model. The authors used a mixed treatment comparisons^d^ framework to synthesise evidence. This study [16] indicated that more complex interventions (which include multiple components such as education, equipment and its fitting, and home inspection) have higher probability of increasing the possession of functioning smoke alarms than those less multifaceted. Nevertheless the authors discussed a series of limitations of this analysis, which included: i) the unavoidable existence of some degree of ‘lumping’ of interventions given existent data; ii) the heterogeneous quality of the evidence base; and iii) the existence of some unexplained inconsistency between direct and indirect evidence.

This paper showed that for these interventions to be adopted, decision makers need to be ‘willing to pay’ or displace large amounts of funds. The less complex intervention of Strategy (3) E + FE was identified to have the lowest ICER when compared to usual care (ICER of £34,200 per QALY gained reducing to approx. £4,500 when 1.8 children under the age of 5 assumed per household).

Four studies to date have conducted cost-effectiveness analysis of smoke alarm interventions [12-15]. Two of these studies were UK based [13,14] and evaluated the provision and installation of free smoke alarms versus ‘no intervention’. The results from the analysis by Pitt *et al.*[14] informed the NICE public health guidance on the prevention of unintentional injuries among under-15s in the home [24]. Our study extends the remit of the previous analyses by considering the cost-effectiveness of multiple interventions (i.e. ranging from usual care to more complex interventions comprising a combination of education, free or low cost equipment giveaway, equipment fitting and/or home safety inspection) to increase the installation of functioning smoke alarms in households with young children. This has been achieved by incorporating effectiveness results from a mixed treatment comparison into the cost-effectiveness analysis – which, to the authors’ knowledge, is the first time that this has been done within a public health study. Our analysis also undertakes a number of sensitivity analyses to test the robustness of the findings to assumptions made by the model. These analyses support the finding of our main analysis that more effective but more complex interventions may not necessarily be the most cost effective interventions.

Where uncertainty over adopting a particular intervention based on existing information exists, the expected consequences of this uncertainty can be quantified. This informs the decision maker of the consequences for the public sector (in £s) of the possibility of making the wrong decision, and informs the maximum value of conducting further research to reduce and improve decision making. In our analysis this was quantified to be approx. £49,900 at a cost effectiveness threshold of £30,000^e^[18]. The decision maker should consider conducting new research only if the costs of the research are lower than this value.

At the basis of the analyses conducted in this study there are a range of limitations. These include, firstly, the difficulty in categorising some of the interventions reporting in the effectiveness studies due to inadequate descriptions of the interventions; for example, education in the different studies may have been of varying intensity. Secondly, although the impact on the results of changing many of the assumptions made in the modelling were investigated in the sensitivity analyses undertaken, not all assumptions were able to be investigated; for example, there is some evidence that a child admitted to hospital with a burn is more likely to be admitted in the future with another burn than with another injury [25]. Thirdly, we know social inequalities exist in the possession of ‘functioning’ smoke alarms in families with children under 5 in the UK and therefore future research may investigate whether more complex interventions may be more cost effective in some social groups [26]. Finally, data on burn treatment costs is country specific; therefore, the results from this analysis (based on UK data) may not necessarily be generalizable to other countries of different healthcare systems.

While economic evaluation has been widely used in the past two decades to support decision making in the health care setting, its use has only recently been applied within public health [20,27-29]. Methodological challenges specific to public health include: (i) the attribution of effects (both intended and unintended) of the policy on the targeted population and problem; (ii) the costs and consequences which should be analysed, considering the feasibility of the programme; (iii) the acceptability of the policy by the relevant stakeholders, which often involves subjective judgements, beliefs, values and interests of the actors concerned, and iv) obtaining an equilibrium between an efficient and an equitable allocation of resources [30,31]. In our analysis we chose a Public Sector perspective, however, if we restricted the analysis to the NHS and PSS (i.e. focusing on healthcare related costs and omitting law enforcement, and Fire and Rescue costs) the ICER for Strategy (3) E + FE, marginally increased from £34,200 to £35,561 per QALY. If we expand the perspective to include property damage, cost of fatality (i.e. coroners, autopsy) and cost of equipment incurred by individual households but not lost productivity costs, then the ICER for Strategy (3) E + FE substantially increased to approx. £74,000 per QALY.

In this paper important findings were made about the cost effectiveness of interventions in promoting the uptake of ‘functioning’ smoke alarms and consequently, in reducing child injuries at home. However, there continues to be insufficient evidence to inform and support public health policy/decision making. This state of affairs can be changed, but it will require strong direction to ensure the priorities for economic evaluation evidence become organised and coordinated at local, regional and national levels.

## Conclusions

• This paper assesses the cost effectiveness of a variety of interventions , ranging from usual care to more complex interventions comprising a combination of education, free or low cost equipment giveaway, equipment fitting and/or home safety inspection;

• Education and free/low cost equipment was identified as the most cost-effective intervention with an estimated ICER of approx. £34,000 per QALY gained compared to usual care;

• Assuming 1.8 children (rather than 1 child) under the age of 5 per household reduces the ICER of the strategy including education and free/low cost equipment to £4,500 per QALY gained compared to usual care.

## Endnotes

^a^‘Functioning’ implies that the safety device is fully operational.

^b^A severe fire-related injury was defined as one that requires inpatient stay greater than five days in an intensive care unit. It was assumed that any child suffering a severe injury (particularly burns) would suffer some form of disability and would carry that impairment for the rest of its life. A child experiencing this event would therefore suffer a decrement in (health related) quality of life and would be subject to additional health costs for the rest of its lifetime. A minor or moderate fire-related injury is assumed not to have any significant decrement in children’s quality of life or any additional on-going health costs.

^c^Note that a predefined threshold does not exist outside of the health sector and therefore the £20,000 to £30,000 range of values is occasionally used throughout to support the interpretation of results.

^d^Mixed treatment comparisons (also known as network meta-analysis) [32-35] are an extension of standard (pairwise) meta-analysis that enable the simultaneous comparison of all evaluated interventions within a single coherent analysis.

^e^Intervention time horizon is assumed to be of 10 years and the annual effective population (i.e. expected number of single child households under 5 per year in the UK) considered is approx. 31,000 (ONS 2010).

## Abbreviations

CODA: Convergence diagnostic and output analysis; E: Education; E + FE: Education + free/low cost equipment; E + FE + HI: Education + free/low cost equipment + home safety inspection; E + FE + F: Education + free/ low cost equipment + fitting; E + HI: Education + home safety inspection; E + FE + F + HI: Education + free/low cost equipment + fitting + home safety inspection; HRQL: Health related quality of life; ICER: Incremental cost-effectiveness ratio; MC: Monte Carlo; MCMC: Markov chain Monte Carlo; NHS: National Health Service; NICE: National Institute for Health and Clinical Excellence; PSS: Personal social care services; QALY: Quality adjusted life years; UC: Usual care; UK: United Kingdom.

## Competing interests

The authors declare that they have no competing interests.

## Authors’ contributions

PS designed and implemented the decision analytic model, conducted the cost-effectiveness analysis and prepared the final manuscript. NJC designed and implemented the decision analytic model, oversaw and advised on all elements of the cost effectiveness analysis and prepared the final manuscript. AJS and DK conceived of the study, helped to develop and populate the decision analytic model, assisted with interpretation of the results and reviewed the final manuscript. MH and KD advised on the model structure, supplied data to inform the model parameters and reviewed the final manuscript. AM advised on elements of the cost effectiveness analysis and reviewed the final manuscript. All authors read and approved the final manuscript.

## Authors’ information

Pedro Saramago and Nicola J Cooper are joint first authors.

## Pre-publication history

The pre-publication history for this paper can be accessed here:

http://www.biomedcentral.com/1471-2458/14/459/prepub

## Supplementary Material

Additional file 1: Table S1 General base-case model inputs. **Table S2.** Base-case model inputs for quality of life weights and costs (updated to 2012 prices) [36]–[51]. Click here for file
