# Supplementary material for: Cost-effectiveness of interventions for increasing the possession of functioning smoke alarms in households with pre-school children: a modelling study
Source: BMC Public Health. 2014 May 16;14:459. doi: 10.1186/1471-2458-14-459 (PMC4046996; doi:10.1186/1471-2458-14-459)
Supplement: Additional file 1: Table S1 — General base-case model inputs. Table S2. Base-case model inputs for quality of life weights and costs (updated to 2012 prices) [36]–[51]. [file 1471-2458-14-459-S1.doc]

**Additional file 1**

**Table S1: General base-case model inputs**

|  | **Point estimate (Standard Error or 95% Credibility Interval)** | **Parameter distribution** | **Source of information** |
| --- | --- | --- | --- |
| **STAGE 1: INTERVENTION MODEL** |  |  |  |
| **Cohort settings** |  |  |  |
| Total number of households in the UK | 26,442,100 |  | 2011 UK Census |
| **Probabilities of possessing a functioning smoke alarms following each intervention:** |  |  |  |
| (1) Usual care | 0.695 (95% CrI: 0.647 to 0.740) |  |  |
| (2) Education | 0.671 (95% CrI: 0.207 to 0.942) |  |  |
| (3) Education + free or low cost equipment | 0.876 (95% CrI: 0.459 to 0.986) |  |  |
| (4) Education + free or low cost equipment + home safety inspection | 0.852 (95% CrI: 0.448 to 0.983) | Posterior distribution inputted directly from MTC analysis | Mixed treatment comparison (MTC) by Cooper *et al.* |
| (5) Education + free or low cost equipment + fitting | 0.859 (95% CrI: 0.400 to 0.982) |  |  |
| (6) Education + home safety inspection | 0.880 (95% CrI: 0.413 to 0.991) |  |  |
| (7) Education + free or low cost equipment + fitting + home safety inspection | 0.941 (95% CrI: 0.651 to 0.993) |  |  |
| **Smoke alarm** |  |  |  |
| Probability accepting intervention (assumed same for all interventions) | 0.9 | Fixed | Assumption based on studies included in NMA |
| Probability a household having a functioning smoke alarm (baseline) | 0.860 | Binomial (n=18,386 sample) | Fire Statistics 2012 (Table 2.3) |
| Probability own a smoke alarm with battery life of 1 year | 0.750 | Binomial (n=15,850 sample) | Survey of English Housing 2004/5 (Table 5.3) |
| Probability test smoke alarm at least once a year | 0.850 | Binomial (n=18,372 sample) | Survey of English Housing 2004/5 (Fig 5.1) |
| Probability test smoke alarm less than once a year | 0.02 | Binomial (n=18,372 sample) | Survey of English Housing 2004/5 (Fig 5.1) |
| **STAGES 2 and 3: PRE-SCHOOL and LONG-TERM MODEL** | | | |
| **Probability of a fire** |  |  |  |
| Probability of a fire where functioning smoke alarms present | Fires where smoke alarm was present, operated and raised or not the alarm = 20,706 (out of 43,451 fires); Assuming that fires occurred in different dwellings:  20,706/26,442,100 = 0.000783 | Binomial (N=26,442,100) | Fire Statistics 2012 (Table 2.4) |
| Probability of a fire where non-functioning smoke alarms present | Fires where smoke alarm was present but did not operate = 7,854 (out of 43,451 fires); Assuming that fires occurred in different dwellings:  7,854/26,442,100 = 0.000297 | Binomial (N=26,442,100) | Fire Statistics 2012 (Table 2.4) |
| Probability of a fire where no smoke alarms present or unspecified | Fires where smoke alarm was absent or unspecified = 14,891 (out of 43,451 fires); Assuming that fires occurred in different dwellings:  14,891/26,442,100 = 0.000563 | Binomial (N=26,442,100) | Fire Statistics 2012 (Table 2.4) |
| Probability of inside household fire being  attended by the Fire and Rescue Service | 0.15 | Binomial (n=272 sample) | Survey of English Housing 2004/5 (Table 3.4) |
| **Probability of injury or fatality** |  |  |  |
| Probability of a fatality following a fire where functioning smoke alarm present | Fires where smoke alarm was present, operated and raised or not the alarm and there were fatal casualties = 122 (out of 287 casualties): 122/20,706 = 0.005892 | Binomial (n=20,706 sample) | Fire Statistics 2012 (Table 2.4) |
| Probability of a fatality following a fire where non-functioning or no smoke alarm | Fires where smoke alarm was present but did not operate or was absent and there were fatal casualties = 165 (out of 287 casualties); 165/(7,854+14,891) = 0.007254 | Binomial (n=22,745 sample) | Fire Statistics 2012 (Table 2.4) |
| Probability of no injury following a house fire with ‘functioning’ and ‘no/non-functioning’ smoke alarms | Probability of injury when functional smoke alarm present = 0.11, therefore the probability of no injury with ‘functioning’ smoke alarm: 1-0.11 = 0.89  Probability of injury when functional smoke alarm absent = 0.125, therefore the probability of no injury without ‘functioning’ smoke alarm: 1-0.125 = 0.875 | Binomial (n=43,451 sample) | Istre *et al.* (Table 2) |
| Probability a child aged 0-4 incurs a minor, moderate or severe injury given a burn injury, following a house fire | minor injury: 0.368  moderate injury: 0.158  severe injury (requires inpatient stay greater than five days in an intensive care unit): 0.474 | Multinomial (n=19 sample) | Personal communication |
| The additional proportion of burn unit costs incurred in ITU | 0.4 (Assumption SE=0.1) | Beta (alpha=9.2, beta=13.8) | Assumption based on analysis in Hemington-Gorse *et al .* |
| Probability have a precautionary check-up following a fire | 0.437 | Binomial (n= 12,935 sample) | Fire Statistics 2007 (Table 8) |
| Probability of all-cause mortality for a UK citizen from 0 to 100 years old (for use in each decision model cycle) | Age dependent |  | Office for National Statistics 2009 |

**Table S2: Base-case model inputs for quality of life weights and costs (updated to 2012 prices)**

|  | **Point estimate (Standard Error or 95% Credibility Interval)** | **Parameter distribution** | **Source of information** |
| --- | --- | --- | --- |
| **STAGE 1: INTERVENTION MODEL** |  |  |  |
| **Intervention costs** |  |  |  |
| Cost of home safety inspection based on cost of local authority home care worker for 40 minutes of their time including travel | (£23/hour, thus 40min =)  £15.33 | Fixed | PSSRU 2012 |
| Cost of smoke alarm giveaway | £4.89 | Fixed | Personal communication |
| Cost of providing education programme per household accepting intervention - based on cost of home care worker for 20 minutes of their time including travel | (Assuming £20/hour, thus 20min =)  £6.66 | Fixed | Assumption |
| Fixed cost of an intervention scheme – programme coordination | Considering a simulated cohort of 100,000 households:  £79,529 | Fixed | DiGuiseppi *et al.* – updated to 2012 prices |
| Additional cost administrative incurred for each household that accept intervention | Distribution costs divided by the number of households in the cohort and updated to 2012 prices = £0.40 | Fixed | DiGuiseppi *et al.* – updated to 2012 prices |
| Cost of having the smoke alarm installed | Installation costs divided by the number of smoke alarms installed and updated to 2012 prices = £11.83 | Fixed | DiGuiseppi *et al.* – updated to 2012 prices |
| **STAGES 2 and 3: PRE-SCHOOL and LONG-TERM MODEL** | | | |
| **Healthcare costs / resource use** | |  |  |
| Mean number of minutes of Paramedic Unit – assumed only attend where severe injuries | 49.5 | Normal (Var = 26.32 (assumption)) | PSSRU 2008 |
| Mean number of minutes of Emergency Ambulance – assumed only attend where moderate injuries | 38.6 | Normal (Var = 26.32 (assumption)) | PSSRU 2008 |
| Cost per minute of a Paramedic Unit | 8.00 | Fixed | PSSRU 2008 – updated to 2012 prices |
| Cost per minute of an Emergency Ambulance | £7.89 | Fixed | PSSRU 2008 – updated to 2012 prices |
| Mean cost (and standard error) of a minor injury | £1,206 (SE = 209) | Lognormal | Personal communication |
| Mean cost (and standard error) of a moderate injury | £2,855 (SE = 1,415) | Lognormal | As above |
| Mean cost (and standard error) of a severe injury | £64,939 (SE = 32,019) | Lognormal | As above |
| Mean incurred NHS costs of disability per year | £379.5 (SE = 85.5) | Gamma (alpha =16, beta=0.047) | HALO study – updated to 2012 prices |
| Cost of precautionary check-up | £68.8 (SE = 21.5) | Normal | NHS reference costs 2008/9 |
| **Out of pocket / private costs** |  |  |  |
| Cost of smoke alarm 1 year battery to individual | £1.54 | Fixed | www.safelincs.co.uk |
| Total cost of damage caused by the fire | £1,298 (SE = 245) | Gamma (alpha=16, beta=0.016) | British Crime Survey: Fires in the Home 2002/3 (Table 3.8) - updated to 2012 prices |
| Cost of a fatality following a household fire – includes coroners and autopsy costs | £205.5 | Fixed | Ginnelly *et al.* (Table 1) – updated to 2012 prices |
| **Law enforcement and rescue services costs** |  |  |  |
| Cost of police attending – assumed only to attend where severe injuries | £173.9 | Fixed | Ginnelly *et al.* (Table 1) – updated to 2012 prices |
| Cost of Fire and rescue Service attending a domestic fire | £3,386 | Fixed | Economic Cost of Fire 2004 (Table 3.6) -– updated to 2012 prices |
| **Utility parameters per cycle** |  |  |  |
| Deficit of utilities for minor injury (DRG 460 + 459) | 0.049 | Fixed | Sanchez *et al.* |
| Deficit in utilities for moderate injury (DRG 458 + 457) | 0.069 | Fixed | Sanchez *et al.* |
| Deficit in utilities for severe injury (DRG 472) | 0.107 | Fixed | Sanchez *et al*. |
| Deficit in utilities following a disability | 0.1 (SE = 0.025) | Beta (alpha = 14.3, beta = 128.7) | HALO study |
| General background utilities for non-injured population | Under 25yrs 0.94 (sd=0.12)  25-34yrs 0.93 (sd=0.15)  35-44yrs 0.91 (sd=0.16)  45-54yrs 0.85 (sd=0.25)  55-64yrs 0.80 (sd=0.26)  65-74yrs 0.78 (sd=0.26)  >75yrs 0.73 (sd=0.27) | Normal | UK Population Norms |
